# Student-perceived exam difficulty may trump the effects of different quality improvement measures regarding the students’ evaluation of a pediatric lecture series

**DOI:** 10.1186/s12909-019-1654-3

**Published:** 2019-06-13

**Authors:** Marco S. Spehl, Christine Straub, Andrea Heinzmann, Sebastian F. N. Bode

**Affiliations:** Center for Pediatrics – Department of general pediatrics, adolescent medicine, and neonatology, Medical Center – University of Freiburg, Freiburg, Germany

**Keywords:** Medical education, Lecture, Evaluation, Peer-lecturer coaching, Audience response system

## Abstract

**Background:**

Lectures are still an important part of today’s medical education at many medical schools. The pediatric lecture series at the Center for Pediatrics, Medical Center, University of Freiburg, Faculty of Medicine, University of Freiburg, Germany had been evaluated poorly in recent terms.

**Methods:**

To improve lecture quality and possibly evaluation results a combination of measures consisting of peer lecturer coaching, use of an audience response system, in depth analysis of the end of term evaluation results and changes to the exam itself were implemented.

**Results:**

Peer lecturer coaching was performed successfully and both the audience response system evaluation as well as the end of term evaluation results improved significantly in the following term. Analysis of the students’ comments revealed more approval of lecture content and presentation after the organization of the lecture series was changed towards less lecturers and focus on less topics. Student-perceived high exam difficulty influenced the evaluation negatively.

**Conclusion:**

The student-perceived exam difficulty can supersede the effects of different measures to improve lecture quality measured via evaluation. Whether better evaluation of the lecture series after different improvement measures was due to better match of the curriculum with the exam content or that an improved curriculum led to better exam performance remains to be elucidated.

## Background

Lectures are still an important part of today’s medical education in many medical schools [1]. Over the last years the lecture series in pediatrics at the Center for Pediatrics, Medical Center, University of Freiburg, Germany (Zentrum für Kinder- und Jugendmedizin, ZKJ) was evaluated poorly in the end of term students’ online evaluation and the exam difficulty was rated as high.

In previous years efforts had already been made at the ZKJ to improve teaching quality in lectures: implementation of an audience response system (ARS) [2, 3], launching of podcasts [4, 5], and lecturer instructions regarding presentation slides as well as live patient presentations during lectures. None of the above significantly and long-lastingly improved the evaluation score in the end of term students’ evaluation or the poor rank in the faculties’ overall department ranking.

Personal lecturer coaching after student evaluation has shown positive effects on teaching quality [6, 7]. Furthermore, positive effects on teaching quality of an assessment of students’ evaluation via free-text commentaries have been shown [8].

The project presented here investigates whether a strategy of a combination of measures including a peer lecturer coaching, exploration of students’ evaluation and improvements in exam organization would lead to improvements in the pediatric department lectures’ teaching quality and student evaluation.

## Methods

**Fig. 1:**
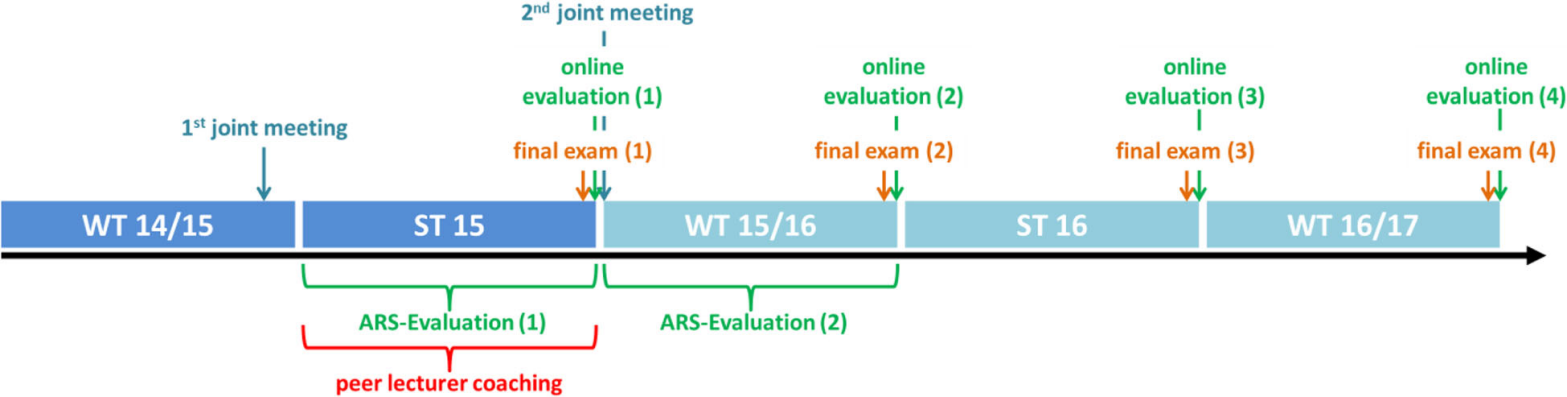
Chronological order of different events and measures from winter term 2014/15 (WT 14/15) until winter term 2016/17 (WT 16/17). Peer lecturer coaching and audience response system (ARS) evaluations were performed after each lecture during the ST 15 and WT 15/16. The final end of term exam was followed by the online evaluation each term (# 1–4). Changes to the lecture plan, learning objectives and the final exam were implemented after the 2nd joint meeting in summer term (ST) 15, therefore WT 15/16 is displayed in a different color. No further changes were implemented in the following terms. Details regarding the changes can be found in the text

To identify areas of possible improvement in the pediatric lectures’ quality at the ZKJ Freiburg a joint meeting of stakeholders including the head of the department, lecturers and consultants responsible for student affairs, who are all involved in teaching at the ZKJ Freiburg, took place in winter term 2014/15. It was decided that the following steps (see Fig. 1) had to be performed:

1. During summer term (ST) 2015: Evaluation of each lecture including a peer lecturer coaching of each lecturer through a peer (MSS or SFNB of the authors).
2. During ST 15 and winter term (WT) 15/16: Obtaining students’ feedback through an audience response system (ARS) at the end of each lecture.
3. After ST 15 and WT 15/16: An in depth analysis of the end of term online open-ended questions evaluation to highlight specific students’ suggestions. Analysis of the overall exam results.
4. After ST 15: Joint meeting to present the data. Hereafter a plan of changes for the schedule of WT 15/16 should be implemented.
5. Evaluation of students’ exam grades and students end of term evaluation in ST 16 and WT 16/17.

### Peer evaluation and lecturer coaching

Each lecturer was informed prior to the start of the term that a peer lecturer coaching would be available. Constructive peer feedback by MSS or SFNB of the authors, who both have undergone intensive training in medical education and didactics, concerning the quality of each lecture, graded on a Likert scale from 1 = very good to 6 = deficient) and a detailed feedback on the structure, visualization, presentation as well as additional free feedback regarding the lecture rated on a standardized peer lecturer feedback form was offered instantly after the lecture. Feedback on lecture structure included, amongst others, rating of the quality of the introduction, educational objectives, adequate amount of data, links to previous knowledge, adequate time management, and quality of the summary/take home messages. Visualization of the lecture consisted of adequate amount of presentation slides, adequate design of the slides (including corporate identity), clear arrangement of the slides, clear rules regarding amount of text on one slide. Feedback on presentation focused on adequate language and speed of speech as well as volume, enthusiasm for the topic, eye contact with the audience, interaction with the audience including asking and allowing questions during the lecture. Additional feedback regarding individual strengths and areas for improvement, one personal highlight, and an overall score was included.

### ARS evaluation

The Power Vote© ARS voting system (La Générale Multimédia, Clichy, France) was used immediately after each lecture in both WT and ST as described in [2]. The students were asked to give their overall rating after each lecture on a Likert scale from 1 = very good to 6 = deficient.

### Analysis of the online end of term evaluation

All students registered for pediatric lectures were invited every term via email by the center for student affairs of the medical faculty, University of Freiburg, to participate in a voluntary and anonymous end of term online evaluation. The evaluation consisted of one Likert scale question and two open ended questions: “How do you rate the quality of the lectures in general?” (Likert scale from 1 = very good to 6 = deficient); “What did you notably like during the lecture series?” (open ended); “Where do you see potential for further improvement during the lecture series?” (open ended).

The open-ended comments were independently and entirely read by two authors (MSS, SFNB) and categories to fit the students’ feedback were deduced according to the literature [8].

### Exam preparation

Exams were prepared using the “Item Management System” (www.ucan-assess.org [9]), a secure online database for exam questions. A combination of questions stored in the database and new questions provided by lecturers was used by MSS or SFNB to compile the exam. The resulting exam questions were checked, corrected and further discussed and adapted during a panel meeting with the heads of the departments for general pediatrics, pediatric cardiology, neuropediatrics and pediatric hematology/oncology.

### Statistical analysis

Quantitative data were analyzed with IBM SPSS Version 20 (IBM SPSS Statistics for Windows, Version 20.0. Armonk, NY: IBM Corp.).

### Ethics

All data on student’s evaluations were only available in anonymized form. All participants of the peer lecturer teaching gave verbal consent to participate, which was recorded with a box to tick on the peer lecturer coaching form, and data are presented in anonymized form. Therefore ethics approval was waived by the ethics committee of the University of Freiburg, Freiburg, Germany.

## Results

### General data and peer lecturer coaching

During ST 15, 28 lectures on pediatric topics were held by 20 different lecturers. 146 students were registered for the course but attendance was not compulsory. One live patient presentation was scheduled every week.

23/28 lectures were attended and evaluated by either MSS or SFNB in ST 15. Due to time constraints (ward duties, night shifts, etc.) the other 5 lectures could not be attended. All but one lecturer agreed to receive a peer lecturer coaching (*n* = 19). The lecturers happily received the peer lecturer coaching but also valued the student’s ARS rating. Most lecturers though rated the peer lecturer coaching as more helpful than the mere ARS evaluation as it provided a more in-depth analysis. 14 of the 19 lecturers who had received a peer lecturer coaching in ST 15 held at least one lecture in the following WT.

During WT 15/16, 28 lectures on pediatric topics were held by 14 different lecturers. 157 students were registered for the course and attendance was not compulsory. One live patient presentation was scheduled every week.

No further changes were implemented in the following two terms. Lecture topics and quantity stayed identical but some few lecturers varied within their specialist’s field.

### Evaluation of the open-ended end of summer term evaluation questions and implementation of changes

The evaluation’s open-ended commentaries were sorted and analyzed as described by [8]. Four categories were approved: “exam”, “lecture style”, “(dis-)likes of specific lectures” and “general aspects”. Feedback was omitted if it included foul language only and did not contain any constructive criticism or feedback at all. The comments were separated from the outset into positive (e.g.: “In my opinion the exam questions were much fairer than in the years before, but still on a pretty high level considering the fact that it’s “only” pediatrics and a very broad field” (Item: fair questions)) and negative (e.g. “The exam tested knowledge that had not been taught in the lectures. I learned quite a lot but still felt poorly prepared and I had a lot of trouble with the questions in the exam. It’s a shame!” (Items: lectures do not prepare well for the exam; very hard exam) feedback.

In a second step the comments were entirely re-read by the two authors and data were coded using words as units of analysis [10]. The (re-)occurrence of each item was tabulated (see results and Tables 1 and 2).

**Table 1:** Summary of the positive feedback taken from the students’ online open-ended questions term evaluation

Positive feedback from the student’s online evaluation						
	ST 15	*N* = 90	WT 15/16	*N* = 86	WT 16/17	*N* = 97
		*n* = 27 (30%)		*n* = 24 (30%)		*n* = 33 (34%)
		Items = 48		Items = 45		Items = 34
Exam						
single choice instead multiple choice	2	4%	0	0%	0	0%
exam procedure/organization	1	2%	0	0%	0	0%
fair questions	1	2%	6	13%	1	3%
elaborate questions	1	2%	0	0%	0	0%
Lecture						
live patient presentations	12	25%	12	27%	7	21%
instructive case examples	2	4%	2	4%	1	3%
MC questions via ARS during lecture	1	2%	0	0%	0	0%
well structured powerpoint slides	2	4%	2	4%	4	12%
lecturer preparation	1	2%	1	2%	3	9%
Likes of specific lectures						
Cardiology	11	23%	5	11%	5	15%
Oncology	2	4%	4	9%	2	6%
Pulmonology	2	4%	1	2%	1	3%
Neonatology	1	2%	2	4%	1	3%
Endocrinology	2	4%	1	2%	0	0%
Neurology	3	6%	0	0%	1	3%
Infectiology	0	0%	2	4%	0	0%
General pediatrics	1	2%	2	4%	2	6%
General aspects						
variation of different topics	1	2%	0	0%	1	3%
script (only neurology)	2	4%	2	4%	3	9%
relevance for professional practice	0	0%	3	7%	2	6%

Comparison between summer term 15 (ST 15), winter term 15/16 (WT 15/16), and WT 16/17. N = number of students participating in the evaluation, n = number of positive comments, Items = number of items within positive statements, i.e. one statement could contain several items e.g. regarding “exam” and “likes of specific lectures”

**Table 2:** Summary of the negative feedback taken from the students’ online open-ended questions term evaluation

Negative feedback from the student’s online evaluation						
	ST 15	N = 90	WT 15/16	N = 86	WT 16/17	N = 97
		*n* = 59 (65%)		*n* = 29 (34%)		*n* = 38 (39%)
		Items = 140		Items = 38		Items = 58
Exam						
vague phrasing of questions	3	2%	0	0%	4	10%
very hard exam (questions)	9	6%	2	5%	8	19%
unclear learning objectives	17	12%	4	11%	1	2%
Lecture						
too little relevant topics	25	18%	3	8%	5	12%
lectures do not prepare well for exam	29	21%	6	16%	15	36%
live patient presentations often cancelled	5	4%	0	0%	4	10%
poor powerpoint layout	6	4%	1	3%	7	17%
lecture content contradicts other lectures	3	2%	0	0%	0	0%
some lecture topics are redundant	4	3%	0	0%	0	0%
too much lecture content	3	2%	1	3%	0	0%
lecture content outdated	1	1%	0	0%	0	0%
need for more lectures	4	3%	0	0%	0	0%
too many lectureres	5	4%	0	0%	0	0%
lecture time inadequate	3	2%	5	13%	7	17%
Dislikes of specific lectures						
Genetics*	6	4%	n.a.		n.a.	
Infectiology	0	0%	1	3%	0	0%
General aspects					0	0%
need for general pediatrics script	4	3%	5	13%	2	5%
need for podcasts of lectures	11	8%	8	21%	5	12%
too much evaluation	1	1%	0	0%	0	0%
poor ARS evaluation questions	1	1%	2	5%	0	0%

Comparison between summer term 15 (ST 15) and winter term 15/16 (WT 15/16). N = number of students participating in the evaluation, n = number of negative comments, n.a. = not applicable, Items = number of items within negative statements, i.e. one statement could contain several items e.g. regarding “exam” and “lecture”. *: the genetics lecture was only held in ST15

The positive list (Table 1) provided only limited data, mostly pointing out popular lectures and the fact that the students valued live patient presentations.

The negative list (Table 2) pointed out that the students were mostly unhappy with the exam: Nine (15%) stated that the questions were too difficult, three (5%) found the phrasing confusing and 17 (29%) were unhappy that there were no clear learning objectives to help them prepare efficiently for the exam. The main complaint in the lecture section was that the lectures did not prepare the students thoroughly enough for the final exam (29 students; 49%). Twenty-five (42%) felt that more relevant topics (e.g. basic facts for daily practice) should be taught. Six (10%) did not like the presentations’ layout or found it ineffective for revision. More details can be found in Table 2.

Lectures that were specifically critized were again reviewed (both lecture slides and results from peer coaching), lecturers were asked to implement changes and some lectures were identified as omittable due to overlapping content or poor assessment in both student and peer coaching evaluation together with a low expectation that suggested changes would be implemented. An example of an omitted lecture is “Pediatric Genetics” for the following reasons: there is another Genetics course within the student’s curriculum covering almost the same lecture content. There was a negative student’s evaluation of the lecture. The respective exam questions were poorly evaluated, most students considered them to be too difficult.

This analysis led to the implementation of the following changes after ST 15:

1. The amount of lecturers was reduced from 20 to 14 in the following term (see above).
2. Five lectures, that were evaluated most poorly by students and peer coaches, were omitted (genetics; pain management; basics of psychosomatics; diet, growth and development; child protection). Two new lecture topics were implemented (pediatric emergencies; diabetes and pediatric diet) because the members of the joint meeting considered them to be both interesting and valuable for the student’s general education and clinical knowledge. A supplementary lecture each to the already existing lectures on gastroenterology, nephrology and infectious diseases was added as peer lecturer coaching identified too much content in these single lectures to ensure enough time to teach basic knowledge.
3. Relevant topics for daily practice were highlighted (e.g. focus on every-day children’s infections rather than focusing on detailed knowledge about orphan diseases in children).
4. The end of term exam was revised: attention was paid to clearer and shorter phrasing and only questions on topics that had been addressed in the lectures were included.

### Comparison between the open ended questions

146 students were enrolled in the ST 2015 pediatric lecture series and 90 (= 61%) students participated in the voluntary evaluation after the end of the term. A total of 157 students were enrolled in the WT 15/16 course, of which 86 (= 55%) participated in the voluntary evaluation after the end of the term. In ST 16 only 15 students participated and the results were not included in this analysis. In WT 16/17 97 (51%) of 189 students participated.

Out of the positive comments it is to highlight that the item “fair exam questions” started from a low 2% (n = 1), rose to 13% (*n* = 6), and then fell again to 3% (*n* = 1) after the last term of the study period. The live patient presentations were equally frequently mentioned in the first two terms but this dropped in the last term as well – looking at the “negative” comment section mentions of missed patient presentations increased from the first to the last term (4 and 10% respectively). See also Table 1 for further results.

The percentage of students’ negative comments dropped almost by half after WT 2015/16 compared to ST 15 (65% vs. 34% respectively) and held steady with 39% after the last term. The items contained within the answers fell dramatically from 140 to 38 and then increased again to 58. An increasing number of students complained about inadequate lecture time, meaning that the lecture took place in the late afternoon between 5 and 6 PM.

After WT15/16 only 5% complained about the exam’s difficulty (6% in ST15, total number decreased from *n* = 9 to *n* = 2) but this rose to 19% (*n* = 8) after WT 16/17. Similar trends could be identified for the item “lectures do not prepare well for the exam”, for more please refer to Table 2.

### End of term online evaluation and ARS evaluation data

In summer term 2015 in total 419 answers of students (m = 19.2, range 7–35) for 26 lectures for 18 different lecturers were recorded by the ARS. Due to technical problems no data were available for two lectures. In winter term 2015/2016 in total 215 answers (m = 10.6, range 1–35) via ARS were given regarding the evaluation of 28 lectures of 14 different lecturers.

Overall, the lecturers in ST 15 received an average grade of m = 2.17 (SD ± .05) (1 = “very good”, 6 = “deficient”) from the attending student’s using the ARS system and an average of m = 1.99 (SD ± .18) from the peer lecturers (p = n.s.). We saw a trend to a correlation between the student’s and the peer lecturer’s overall rating after each lecture (except in one case). The lecturers in WT 15/16 received an average of m = 1.71 (SD ± .06) from the attending students using the ARS (ST 15 vs. WT 15/16: p = < .0001).

No significant changes in the ARS evaluation of individual lecturers could be observed but there was a trend to a better evaluation in WT 15/16 in 13/14 lecturers that held lectures in both terms.

The overall end of term online evaluation improved significantly from m = 3.43 (ST 15; *n* = 90; SD ± 1.2) to m = 2.36 (WT 15/16; *n* = 72, SD ± .78, *p* < .001). This positive trend seemed to hold true in the following term, even though due to technical difficulties only 15 students participated in the end of term evaluation. In WT 16/17 the end of term evaluation resulted in m = 2.88 (*n* = 86, SD ± 1.01). Compared to ST 2015 it was still significantly better (*p* = .002) but also significantly worse than in WT 15/16 (*p* = .0004).

### Exam results

The student’s average exam grade in ST 15 was 2.79 (SD ± .089, *n* = 147) and m = 2.49 (SD ± .077; *n* = 157) in WT 15/16 respectively (*p* = .009). In ST 16 the average exam grade was m = 2.67 (SD ± .82, *n* = 158), and in WT 16/17 m = 2.72 (SD ± 1.03, *n* = 189) – no significant differences to the other average exam grades were found.

## Discussion

This study describes the process of implementing different procedures to potentially improve the quality of a pediatric lecture series as evaluated by students.

With a combination of measures including adapting lecture topics and content, reducing the number of lecturers, standardized and personalized peer-to-peer lecturer coaching with a focus on presentation skills, the use of an audience response system and revision of the end of term exam we were able to achieve a significant improvement in the standardized end of term student evaluation – albeit the effects were not long-lasting.

Due to the implementation of different changes at our medical school at the same time and the design of the study it is impossible to compare the effect of single changes to the overall evaluation. But is it only comparing apples with oranges or can we still learn something from the work presented here? We again have to analyze the different measures implemented in detail.

### Peer lecturer coaching

Most of the lecturers being coached appreciated the detailed and personalized feedback. The coaches could witness the implementation of changes in some lecturers, who read more than one lecture, directly e.g. regarding organization of lecture slides or implementing case studies. Also when looking at the negative feedback provided in the end of term students’ evaluation we noticed a significant drop in negative comments regarding lecture relevance, lecture layout, and lecture content redundancy in the following term, amongst others.

This is in line with reported positive effects of peer lecturer coaching [6, 7] and might contribute to the better overall evaluation to some degree after the peer lecturer coaching. Many of the suggested changes are easy to implement but repeated reminders might be needed to keep up the quality of the lecture series. It would be desirable to be able to provide a peer lecturer coaching on a regular basis every term or at least for some lecturers to ensure sustained effects but due to time and financial constraints this would be a challenge at the ZKJ Freiburg.

### End of term evaluation and ARS evaluation

#### Quantitative evaluation

Positive effects of a well-perceived “performance” of a lecturer have been reported [2, 11, 12]. This might explain some of the differences in the ARS and the end of term evaluation as all students who registered for the pediatric lecture course could participate in the end of term evaluation, even those who never went to a single lecture and therefore could not appreciate any of the didactic methods the lecturers chose. Those same students might have evaluated worse than those who participated in the lectures as actually not all lecture slides were available online and those who did not participate might have been at a disadvantage regarding the exam.

Both ARS evaluation and online end of term evaluation improved significantly after the implementation of the changes so it can be speculated that the positive aspects noted by the students in the lecture and reflected by the ARS data were carried over to some extent to the end of term evaluation – however, those changes were observed only temporarily as well. Additionally we did not observe significant improvements for individual lecturers. The overall better evaluation might be caused by the individual trend we saw in almost all lecturers and the omission of some of the previously most poorly evaluated lectures.

Both ARS and end-of-term evaluation rely on one single item asked which might not be able to show the complete picture – the additional quantitative evaluation might be helpful here.

#### Evaluation of the free-text comments

Rather than looking only at overall quantitative results a detailed analysis of the free text commentaries’ content revealed plentiful improvable aspects and, after changes were applied, the general picture of the commentaries changed towards the positive. Quantitative content analysis has been implemented in the social sciences [8, 10, 13] but in medical education the focus still is on the overall grade of a quantitative evaluation. Analyzing qualitative data can help identify improvable aspects and should be considered - but is time consuming and challenging. Receiving the comments electronically can help save time and allow for easier categorization. The low number of students’ comments must be assessed critically though and improving the student participation rate in free-text evaluation is crucial. This study does show clear changes in the overall regarding measures implemented and identifies possible areas of improvement.

#### Exam

The student-perceived exam difficulty has an influence on evaluation results [14] and plays a major role in this study. The exam at the end of the lecture course was rated easier by the students in WT 15/16 after the implementation of the changes reported as per the free-text commentaries and on the objective side the average exam grade improved - which is consistent with the literature [14]. Even though the average exam grades fell again in the following terms, this was not significant and changes in the end of term evaluation at least stayed significantly different when compared to ST 15, prior to the implementation of the changes of the lecture series. Whether these observations are in fact due to a better match between exam and curriculum content or that the improved curriculum led to better exam performance needs to be the focus of future studies. The average evaluation results dropped significantly after WT 16/17 compared to the term after the implementation of the changes leading to the possible conclusion that the student-perceived exam difficulty plays a major role in the evaluation results given. Even more attention than before will have to be paid in the future to ensure consistent exam difficulty and continuous efforts are needed to ensure consistent lecture quality.

With this study the effect of the exam’s difficulty on the evaluation cannot be quantified exactly. As data from the evaluation were anonymized those could not be matched to individual exam results preventing further analysis. Many aspects that were rated negatively in ST 15 improved significantly after WT15/16 so it is possible that the exam was not the only factor improving the overall rating and that attention should be paid to the other measures presented here as well.

#### Longer term outcome and outlook

The evaluation results seem to hold steady only for a short period of time after a combination of measures to improve the lectures’ quality was implemented.

As peer-lecturer coaching holds significant investment in person-hours it might not be feasible to implement this on a regular basis but only in selected cases. ARS and a detailed analysis of the free-text answers in the student end of term evaluation might help to identify possible problematic lectures that could then be targeted by peer lecturer coaching.

It would also be desirable to conduct the evaluation before the exam to minimize the exam’s influence on the evaluation results – due to tight term schedules this might prove challenging. Other factors e.g. timing of the lecture, availability of patients for live patient presentations in the lectures are demanding to change.

The overall aim of medical education certainly is not to receive good evaluation results, it is to enable students to gain basic medical knowledge – in this case in pediatrics – to be able to examine and possibly treat pediatric patients irrespective of the later specialization the students choose. Fair exams are known to motivate students’ learning [15] and continuous efforts will be put into ensuring that the exams at the ZKJ Freiburg are exactly that: fair but not necessarily easy, and challenging to some students. We believe this is the right approach as students from the University of Freiburg medical school score consistently among the top 5 universities in Germany regarding the results of the final written medical licensing exam and pediatric questions contribute to a significant degree to the exam [16]. We hope that the knowledge the students gained to score that high will be for the benefit of pediatric patients later treated by those students.

We believe that many medical schools may face similar issues in evaluation and hope that our approach might give some ideas on how to improve lecture series or the evaluation of medical education. As our data suggest continuous efforts have to be put into teaching to keep the quality, as rated by students’ evaluation, high.

## Conclusions

A combination of measures to improve a lecture series’ quality can have a positive impact on the overall student evaluation – but different factors contributing to this positive development can be hard to distinguish and the effect might only be temporarily. Considerable time and effort is needed to implement a regular peer lecturers coaching. ARS and analysis of students’ free text commentaries might aid in identifying lectures that would benefit from peer lecturer coaching. The student-perceived exam difficulty seems to have a at least some influence on the evaluation results.

## Data Availability

The datasets used and/or analyzed during the current study are available from the corresponding author on reasonable request.

## Abbreviations

ARS: Audience Response System
e.g.: For example
m: Mean
n: Number (of participants)
SD: Standard deviation
ST: Summer Term
WT: Winter Term
ZKJ: Center for Pediatrics, Medical Center, University of Freiburg, Germany
